# Brain morphometry in hepatic Wilson disease patients

**DOI:** 10.1002/jimd.12814

**Published:** 2024-11-19

**Authors:** Parya Rahimi, Stanislav Mareček, Radan Brůha, Monika Dezortová, Petr Sojka, Milan Hájek, Marta Skowrońska, Łukasz Smoliński, Petr Urbánek, Tomasz Litwin, Petr Dušek

**Affiliations:** ^1^ Department of Neurology and Center of Clinical Neuroscience, First Faculty of Medicine Charles University and General University Hospital in Prague Prague Czechia; ^2^ Fourth Department of Internal Medicine, First Faculty of Medicine Charles University and General University Hospital in Prague Prague Czechia; ^3^ MR Unit, Department of Diagnostic and Interventional Radiology Institute for Clinical and Experimental Medicine Prague Czechia; ^4^ Second Department of Neurology Institute of Psychiatry and Neurology Warsaw Poland; ^5^ Department of Medicine, First Faculty of Medicine Charles University and Military University Hospital Prague Czechia

**Keywords:** brain atrophy, brainstem, hepatic, morphometry, MRI, white matter, Wilson disease

## Abstract

Wilson disease (WD) primarily presents with hepatic and neurological symptoms. While hepatic symptoms typically precede the neurological manifestations, copper accumulates in the brain already in this patient group and leads to subclinical brain MRI abnormalities including T2 hyperintensities and atrophy. This study aimed to assess brain morphological changes in mild hepatic WD. WD patients without a history of neurologic symptoms and decompensated cirrhosis and control participants underwent brain MRI at 3T scanner including high‐resolution T1‐weighted images. A volumetric evaluation was conducted on the following brain regions: nucleus accumbens, caudate, pallidum, putamen, thalamus, amygdala, hippocampus, midbrain, pons, cerebellar gray matter, white matter (WM), and superior peduncle, using Freesurfer v7 software. Whole‐brain analyses using voxel‐ and surface‐based morphometry were performed using SPM12. Statistical comparisons utilized a general linear model adjusted for total intracranial volume, age, and sex. Twenty‐six WD patients with mild hepatic form (30 ± 9 years [mean age ± SD]); 11 women; mean treatment duration 13 ± 12 (range 0–42) years and 28 healthy controls (33 ± 9 years; 15 women) were evaluated. Volumetric analysis revealed a significantly smaller pons volume and a trend for smaller midbrain and cerebellar WM in WD patients compared to controls. Whole‐brain analysis revealed regions of reduced volume in the pons, cerebellar, and lobar WM in the WD group. No significant differences in gray matter density or cortical thickness were found. Myelin or WM in general seems vulnerable to low‐level copper toxicity, with WM volume loss showing promise as a marker for assessing brain involvement in early WD stages.

## 1 INTRODUCTION

Wilson disease (WD) is a rare genetic disorder of copper metabolism caused by bi‐allelic pathogenic variants in the *ATP7B* gene encoding the copper‐transporting ATPase B.^1^, ^2^ The liver, having the highest expression of this protein, is the primary organ responsible for regulating systemic copper balance; therefore, liver injury is the earliest and most frequent manifestation of WD.^3^ When the liver's capacity to buffer excessive copper intracellularly is exhausted, copper is released into the bloodstream and deposits in various organs, particularly the brain, with the basal ganglia and brainstem being the most susceptible brain regions.^4^ The clinical presentation of WD is mainly hepatic or neurologic, with hepatic symptoms typically presenting up to 10 years before the onset of the neuropsychiatric disease characterized primarily by movement disorders and various psychiatric symptoms.^4^

It is expected that in untreated patients gradual copper accumulation in the brain occurs already in the presymptomatic stage. Its excessive brain levels were documented in ex vivo studies in patients with the hepatic form of the disease.^5^ Moreover, structural brain MRI abnormalities attributable to copper deposition, particularly altered T2 signal in the deep gray matter (GM) and brainstem, were detected in ~40% of hepatic and ~25% of presymptomatic patients.^6^ These T2‐hyperintense brain lesions may reflect demyelination, edema, and gliosis, and are at least partially reversible with anti‐copper treatment. In contrast, brain atrophy, a common finding in neurologic WD that correlates with neurologic impairment, is irreversible and represents a promising biomarker for disease monitoring and prognosis.^7^ In hepatic WD, however, brain atrophy is not obvious on standard brain imaging and thus quantitative brain morphometric studies are needed. To date, only few studies have assessed brain volume with such methods in hepatic WD. Viveiros et al.^8^ found evidence of subclinical subcortical brain atrophy involving the cerebellum in a small group of patients with hepatic WD of various severity. However, in that study, hepatic encephalopathy cannot be excluded as a confounding factor. Consequently, whether patients with less severe hepatic WD display brain atrophy remains unclear.

Therefore, this study used quantitative brain morphometric methods to analyze brain volume in patients with mild hepatic symptoms to remove the potential confounding of decompensated liver disease.

## 2 METHODS

### 2.1 Study participants

This was a retrospective study of patients with mild hepatic WD with a variable treatment duration. Patients and healthy controls were enrolled in two centers in Prague, Czechia, and Warsaw, Poland. WD was diagnosed according to the current guidelines,^9^ with either genetic confirmation of two pathogenic variants in the *ATP7B* gene or liver copper content >250 μg/g in liver biopsy in those patients with only one pathogenic variant confirmed. Only patients with well‐compensated hepatic WD manifestation were included, that is, those without a history of liver transplantation, cirrhosis documented on morphological examination, or symptoms of decompensated liver disease, such as ascites or hepatic encephalopathy. Patients with neurologic or psychiatric symptoms of WD were not eligible. We excluded patients with MRI scans that displayed motion artifacts or structural abnormalities affecting morphometric analyses. Healthy controls were recruited from co‐workers and their friends and families and were selected to have age and sex comparable to the patient group. For inclusion, healthy controls had to be free from neurologic or psychiatric disorders and structural lesions on brain MRI.

Medical charts throughout the entire observation period were reviewed, and liver ultrasound findings were retrieved, including evaluation of fibrosis and steatosis (continuous attenuation parameter) staging if transient elastography was performed; in a subset of patients who underwent liver biopsy, histological findings were also recorded. Furthermore, the presence of Kayser–Fleischer ring at the time of diagnosis, assessed by a slit‐lamp examination, was retrieved.

Within 1 month of the MRI scan, patients underwent clinical and laboratory assessment to evaluate hepatic and neurologic function. Neurologic function was assessed using the Unified WD Rating Scale, with part II evaluating activities of daily living and part III assessing abnormalities on neurologic examination.^10^ Liver function was evaluated using the Fibrosis‐4 (FIB‐4) index, a non‐invasive scoring system that estimates the degree of liver fibrosis based on age, platelet count, serum aspartate aminotransferase, and alanine aminotransferase levels^11^ as well as the Child–Pugh score, which assesses the severity of chronic liver disease, particularly cirrhosis, by considering serum bilirubin, albumin, prothrombin time (INR), and the presence of ascites and hepatic encephalopathy.^12^

All information was accessed under the applicable laws and ethical requirements for the study period in compliance with the Declaration of Helsinki revised in 2013. All examined patients signed informed consent. The study was approved by the appropriate Institutional Review Boards.

### 2.2 MRI data acquisition

MRI examinations were performed on 3T scanners and included routine clinical T2‐weighted, susceptibility‐weighted, and magnetization‐prepared rapid gradient‐echo (MPRAGE) images covering the whole brain.

In the Prague center, MRI scans were obtained with the Siemens Trio scanner (Siemens, Erlangen, Germany) with a 12‐channel birdcage head coil. MPRAGE images were acquired using the following parameters: repetition time = 2300 ms; echo time = 4.4 ms; inversion time = 900 ms; flip angle = 10°; field of view = 216 × 256 × 160 mm^3^; voxel resolution = 1 × 1 × 1 mm^3^; bandwidth = 150 Hz/pixel.

In the Warsaw center, the GE SIGNA Architect scanner (GE Medical Systems) equipped with a 48‐channel head coil was used. MPRAGE images were acquired using following parameters: repetition time = 2700 ms; echo time = 3.4 ms; inversion time = 1000 ms; flip angle = 8°; field of view = 230 × 230 × 180 mm^3^; voxel resolution = 0.8 × 0.8 × 0.8 mm^3^; bandwidth = 122 Hz/pixel.

### 2.3 Visual MRI analysis

MPRAGE, susceptibility‐weighted, and T2‐weighted images of patients with WD were visually analyzed by one rater (PD) using the previously validated WD brain MRI severity scale to calculate the acute toxicity and chronic damage indices.^7^ MPRAGE images were specifically examined for an increase in T1 signal in the globus pallidus or other deep GM structures.

### 2.4 Volumetric data processing

The segmentation of brain regions was performed with the Freesurfer software (version 7.3.2),^13^ allowing the identification of 12 distinct regions of interest (ROIs) in the deep GM nuclei and posterior fossa structures known to be affected by WD. These ROIs included nucleus accumbens, amygdala, caudate, cerebellar white matter (WM), cerebellar cortex, hippocampus, midbrain, globus pallidus, pons, putamen, superior cerebellar peduncle (SCP), and thalamus. Subsequently, volumetric measurements were automatically computed. In paired structures, volumes from both hemispheres were summed and all regional volumes were adjusted for total intracranial volume (TIV) using the residual method as previously described.^14^

### 2.5 Whole‐brain voxel‐based and surface‐based morphometry

For voxel‐based morphometry (VBM) and surface‐based morphometry (SBM), the MRI preprocessing was done using the Statistical Parameter Mapping 12 (SPM12; version 7771) and the Computational Anatomy toolbox 12 (CAT12; version CAT12.8.2, r2170).

For VBM, MPRAGE images were normalized and segmented into WM, GM, and cerebrospinal fluid. The brain parenchymal fraction (BPF) was calculated as follows: (GM + WM)/TIV. The segmented GM and WM images were smoothed with a Gaussian kernel of 8 mm full width at half maximum and subsequently used in statistical analysis to assess GM and WM atrophy.

Cortical thickness was estimated according to the earlier published method by Dahnke et al. (2013).^15^ The SBM data were resampled to a 32 000 vertices mesh and smoothed with a Gaussian kernel of 12 mm full width at half maximum.

### 2.6 Statistical analysis

TIV‐adjusted volumetric data were compared between WD and controls using the general linear model with sex, age, and center as covariates of no interest; the Holm–Bonferroni correction for multiple comparisons was applied. To further account for the effect of different scanners in both centers, a sensitivity analysis was performed after processing the volumetric data using the neuroHarmonize software (https://github.com/rpomponio/neuroHarmonize), a data harmonization tool for multi‐site neuroimaging analysis.^16^

Statistical analysis of voxel‐wise methods was performed using the non‐parametric threshold‐free cluster enhancement test with 10 000 permutations; other parameters were set to default.^17^ Age, sex, center, and in the case of VBM also TIV were used as covariates of no interest. A family‐wise error‐corrected *p*‐value <0.05 was used as a threshold for statistical significance.

## 3 RESULTS

### 3.1 Participant characteristics

Of 41 WD patients with hepatic WD and available brain MRI scans, 11 were excluded due to low‐quality MPRAGE scans, two due to structural brain abnormalities (large arachnoid cyst), one due to advanced cirrhosis, and one due to neurologic symptoms of unclear etiology. Thus, 26 WD patients, 10 from the Prague and 16 from the Warsaw center, and 28 controls, 16 from the Prague and 12 from the Warsaw center were included (Table 1). Except for six individuals with only one pathogenic variant, two *ATP7B* pathogenic variants were detected in all WD patients; the H1069Q was the most prevalent variant with an allelic frequency of 64%. Eighteen patients were clinically asymptomatic at diagnosis, with elevated liver function tests being the presenting sign that triggered further evaluation. In seven patients, the diagnostic workup started as family screening. Dyspepsia, abdominal pain, and fatigue were the most common initial symptoms occurring in a total of eight patients. None of the patients had signs of chronic advanced liver disease with significant portal hypertension. Liver morphology by ultrasound examination was normal in seven patients, 14 had steatosis, and four had steato‐fibrosis.

**TABLE 1:** Demographic and clinical characteristics of Wilson disease (WD) patients and controls.

	WD (*n* = 26)	Controls (*n* = 28)
Females/males, *n* (%)	11/15 (43/57%)	15/13 (54/46%)
Age at MRI^a^	29.6 ± 8.9	32.6 ± 9.1
Kayser–Fleischer ring, *n* (%)	2 (8%)	n/a
Presenting symptoms/signs, *n* (%)		
Increased LFT	25 (96%)	n/a
Abdominal pain/dyspepsia	5 (19%)	
Fatigue	4 (15%)	
Anemia	1 (4%)	
Amenorrhea	1 (4%)	
Positive family history, *n* (%)	7 (27%)	n/a
UWDRS part II score^b^	0 (0–0)	n/a
UWDRS part III score^b^	0 (0–1.25)	n/a
FIB‐4 score^a^	0.72 ± 0.51	n/a
Treatment, *n* (%)		
Treatment naïve	3 (12%)	n/a
d‐Pencillamin	11 (42%)	
Zinc salts	11 (42%)	
Combination	1 (4%)	

Abbreviations: FIB‐4, Fibrosis‐4 index for liver fibrosis; LFT, liver function tests; n/a, not available; UWDRS, Unified Wilson Disease Rating Scale.

^a^ Values reported as mean ± standard deviation.

^b^ Values reported as median (interquartile range).

At the time of the MRI, three patients were still treatment naïve, 11 were treated with d‐Penicillamine, 11 with zinc salts, and one with a combination of the two medications. The mean treatment duration was 13 ± (SD) 12 (range 0–42) years. The Child–Pugh score was 5 in all patients indicating Class A liver disease, the mildest form of liver impairment in the Child–Pugh system, with preserved hepatic function. The mean FIB‐4 score was 0.72 (range 0.25–2.60), indicating a low likelihood of significant liver fibrosis in the majority of patients. Only two patients had a FIB‐4 score greater than 1.45, falling into the range of intermediate probability of fibrosis. A comprehensive overview of patient clinical characteristics is presented in Table S1.

### 3.2 Visual and volumetric MRI analysis

No hyperintense lesions were apparent on T1‐ and T2‐weighted images; the acute toxicity MRI score was zero for all patients. The chronic damage MRI score was zero in 11 patients, while 15 scored 1 or 2, mostly due to T2‐hypointense signal in the deep GM nuclei.

Adjusted for age, sex, and center, the mean BPF was significantly lower in patients with WD than controls (0.81 ± 0.04 vs. 0.82 ± 0.03; *p* = 0.005). Compared to controls, patients with WD had smaller volumes of the pons, cerebellar WM, midbrain, and SCP. However, after applying the Holm–Bonferroni correction, only the volumetric difference in the pons remained statistically significant (13.8 ± 1.6 vs. 15.1 ± 1.5 cm^3^; *p* = 0.001) (Table 2). There was no significant interaction between the group and center for any of the analyzed ROIs indicating that the between‐group differences in ROI volumes are consistent across both centers. The mean regional volumes for both centers are shown in Table S2. Complementary sensitivity analysis employing the neuroHarmonize tool to adjust for potential bias related to different MRI scanners showed similar results (Table S3). We conducted an exploratory analysis to assess the potential effect of treatment type, comparing WD patients on d‐Penicillamine (*n* = 11) and Zinc monotherapy (*n* = 11) using values processed by the neuroHarmonize tool. No significant volumetric differences were found between the two treatment groups (Table S4).

**TABLE 2:** Comparison of mean regional volumes in Wilson disease (WD) patients and controls.

	WD	Controls	*p*‐value
Brain parenchymal fraction %	0.81 ± 0.04	0.82 ± 0.03	**0.005**
Nucleus accumbens	1.1 ± 0.2	1.0 ± 0.2	0.77
Amygdala	3.3 ± 0.4	3.4 ± 0.4	0.35
Caudate	7.3 ± 1.1	7.3 ± 0.8	0.31
Cerebellar cortex	113.2 ± 10.4	114.8 ± 8.0	0.09
Cerebellar white matter	26.7 ± 3.6	29.0 ± 3.1	**0.03**
Hippocampus	8.6 ± 0.7	8.4 ± 0.5	0.06
Midbrain	6.1 ± 0.4	6.3 ± 0.5	**0.05**
Globus pallidus	3.9 ± 0.4	4.1 ± 0.3	0.06
Pons	13.8 ± 1.6	15.1 ± 1.5	**0.001**
Putamen	10.1 ± 1.2	9.9 ± 0.9	0.73
Superior cerebellar peduncle	0.2 ± 0.0	0.3 ± 0.0	**0.05**
Thalamus	15.0 ± 1.1	15.4 ± 1.0	0.06

*Note*: Mean values ± standard deviations are reported; except for brain parenchymal fraction all values are shown as total intracranial volume‐corrected volumes in (cm^3^); *p*‐values are adjusted for age, sex, and center; significant differences are marked with BOLD font.

### 3.3 VBM and SBM

No significant interaction between center and group was found in either VBM or SBM analyses. SBM and VBM of the GM revealed no significant differences between WD patients and healthy controls.

**FIGURE 1:**
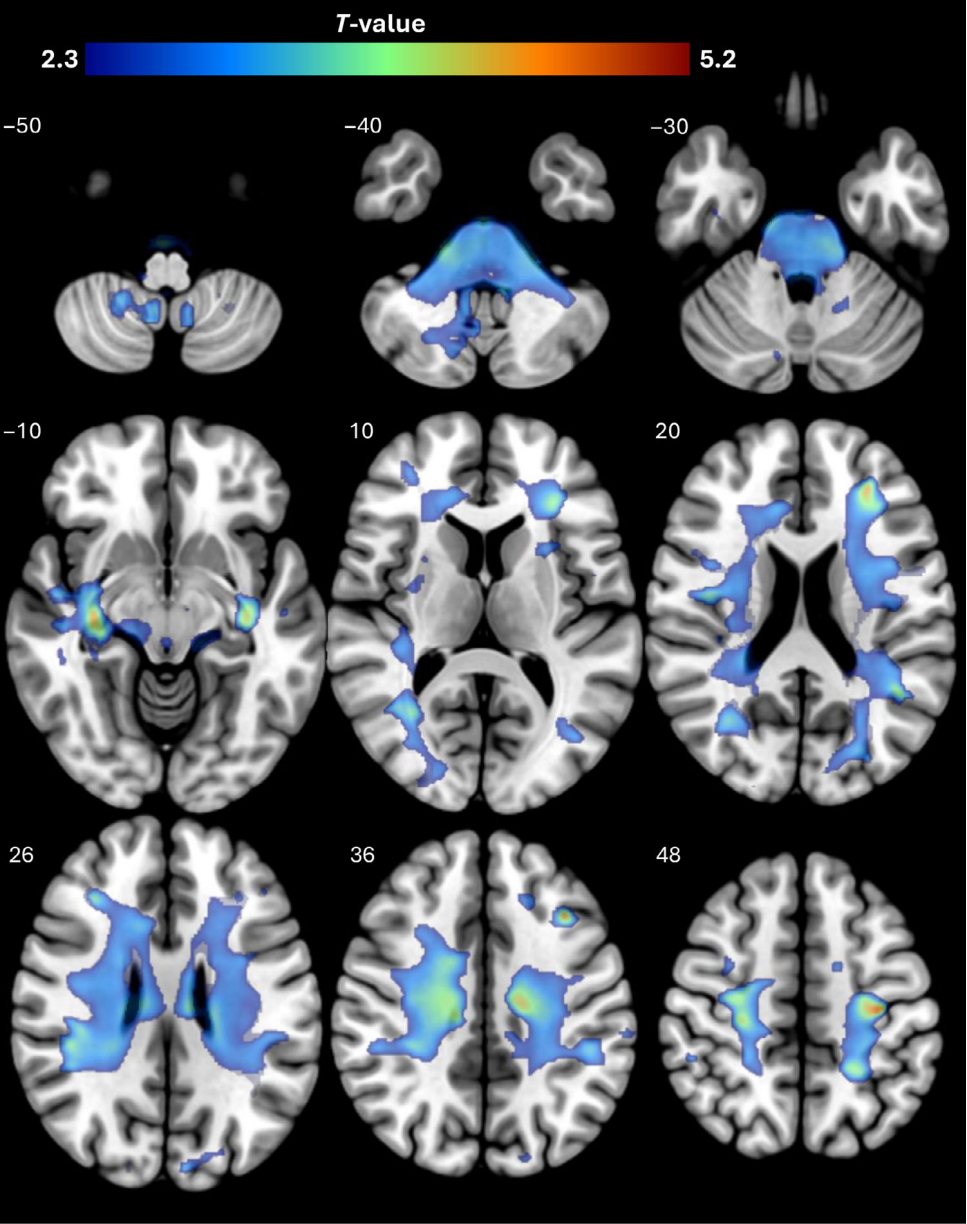
Voxel‐based morphometry results for contrast controls >Wilson disease in the axial plane. *T*‐values of regions with significant volume loss (thresholded at P_FWE_ <0.05) are displayed. *Z*‐coordinates in mm in the Montreal Neurological Institute space are shown for each slice. FWE, family‐wise error‐corrected.

VBM of the WM showed reduced volume in patients with WD in the pons, cerebellar WM, and lobar WM. According to the NatBrainLab WM atlas,^18^ these regions included cortical‐pontocerebellar tracts, SCP, middle cerebellar peduncle, bilateral corpus callosum, cingulum, anterior commissure, arcuate fasciculus, cortico‐spinal tracts, inferior longitudinal fasciculus, occipitofrontal fasciculus, and optic radiations (Figure 1; Table S5).

## 4 DISCUSSION

In this study, patients with mild hepatic WD had a significantly reduced BPF, primarily due to a reduced volume of WM. Regional analyses revealed significantly decreased volumes of the pons, cerebellar, and lobar WM. Overall, our findings suggest a diffuse subclinical involvement of WM in patients with WD and isolated mild hepatic symptoms.

Our results add to the evidence on brain involvement in non‐neurological presentations of WD. Brain MRI findings in hepatic WD are heterogeneous and include T2 hyperintense lesions, brain atrophy, and signs of hepatic encephalopathy, with the severity depending on the degree of cerebral copper accumulation and hepatic dysfunction.^6^, ^8^, ^19^, ^20^, ^21^, ^22^, ^23^, ^24^ Kozić et al.^20^ and Diao et al.^21^ reported T2 hyperintensities in the brains of patients with hepatic WD; in all untreated patients, brain lesions were detected primarily in the basal ganglia, thalamus, and midbrain; among treated patients, 44% had these lesions. Likewise, brain atrophy was noted in 60% of patients with hepatic WD.^21^ Similarly, Litwin et al.^6^ reported brain changes in 40% of treatment‐naïve patients with hepatic WD. Compared to neurologic WD, the hepatic form of the disease was associated with a lower burden of T2 lesions and milder brain atrophy.^7^, ^25^

In our study, a standard visual brain MRI analysis was inconspicuous in all patients documenting their mild severity. Most of the patients were clinically asymptomatic at diagnosis, and the diagnostic workup had been triggered by abnormal liver function tests or a family history of WD. Thus, the patients included in our study were diagnosed at the earliest possible stage of WD barring the introduction of newborn screening into routine practice. Additionally, most of our patients had been on anti‐copper treatment for 2 years or longer, which is typically sufficient to improve reversible T2 hyperintense lesions.

Few studies investigated quantitative MRI in hepatic WD. Our study corroborated previous observations of a significant reduction in the pons and cerebellar and lobar WM volume in these patients.^8^, ^26^ Contrary to previous findings, we did not find thalamic or frontal cortical atrophy, possibly due to the exclusion of patients with advanced hepatic pathology in our study.

Overall, brain pathology in WD can be attributed to hepatic dysfunction or copper toxicity. Prominent structural brain abnormalities are present in advanced liver disorders such as cirrhosis or portosystemic shunts independently of their etiology.^24^, ^27^, ^28^ These brain abnormalities include bilateral T1 hyperintensities in the globus pallidus and pontocerebellar atrophy as reported in a condition termed acquired hepatocerebral degeneration.^29^, ^30^, ^31^ Bilateral T1 hyperintensities in the globus pallidus, which are likely due to the deposition of manganese and tend to disappear with liver function improvement, were also described in patients with WD and hepatic encephalopathy.^22^, ^24^ Moreover, patients with liver cirrhosis due to causes other than WD may show prominent atrophy in the basal ganglia and cerebellum as well as frontal, parietal, and temporal cortex and WM; the extent of atrophy was associated with the severity of hepatic encephalopathy.^32^, ^33^ In contrast, one study in patients with cirrhotic liver disease documented increased WM volume in the internal capsule and cerebellar WM, attributing this finding to low‐grade brain edema caused by astrocytic swelling.^34^

Our study included only patients with mild hepatic WD, whereas those patients with decompensated cirrhosis or hepatic encephalopathy were excluded. Selecting such patients and seeing brain abnormalities that do not resemble those typically associated with liver disease makes us suspect that the morphometric abnormalities in our patients are not driven by hepatopathy but rather by specific WD‐associated pathology. Therefore, the reduction in WM volume observed in our study may be attributed to brain copper accumulation. However, this interpretation does not agree with the observations that basal ganglia are most sensitive to copper toxicity in WD^5^, ^35^; these regions did not show substantial abnormality in our study. Yet, we included patients who started anti‐copper treatment at a very early stage of WD when only limited tissue copper accumulation can be expected. Low extra‐hepatic tissue copper deposits in our patients are also documented by the low prevalence of K–F rings at the diagnosis. We speculate that low‐grade copper deposits may predominantly affect WM structures because of the high sensitivity of myelin to copper toxicity,^36^ whereas the threshold for basal ganglia toxicity may be higher. Of note, a WM volume reduction accompanied by T2 hyperintense lesions, pontine atrophy, and T2 signal abnormalities resembling central pontine myelinolysis were previously reported in both hepatic and neurologic WD with more severe abnormalities observed in the latter.^8^, ^20^, ^21^, ^27^, ^37^, ^38^, ^39^, ^40^, ^41^, ^42^ Thus, WM alterations are not specific to hepatic WD but may indicate an early stage of cerebral injury in WD. Given that ATP7B is expressed in the brain,^43^ dysfunction of this protein may theoretically also contribute to WM volume reduction. This hypothesis could be tested in in vitro or animal studies.

Although patients included in our study were neurologically asymptomatic, detailed cognitive or neurophysiological testing could reveal functional correlates of the morphological abnormalities found in our study. Indeed, specific cognitive deficits associated with WM MRI diffusion metrics were previously observed in WD patients with and without neuropsychiatric symptoms.^19^, ^44^, ^45^ Pontocerebellar pathways are critical for articulation, eye movements, postural balance, sleep, and mood regulation; impairment of these functions is frequently present in neurologic WD.^3^, ^6^, ^8^, ^19^, ^20^, ^21^, ^40^, ^46^ Future studies should investigate the signs of pontocerebellar dysfunction in hepatic WD. Overall, the finding of potentially preventable brain volume loss in patients diagnosed at an early stage supports the inclusion of WD in newborn screening programs. Suitable screening methods based on genetic testing^47^, ^48^ or ATP7B protein detection^49^ are being developed, and efforts should be made to incorporate them into clinical practice.^50^

Our study had limitations. First, because of the low prevalence of mild hepatic WD, we had to include patients from two centers examined with different scanners. Although the effect of center was accounted for, analyzing images from one scanner would be preferable. Second, the retrospective design allowed only a limited assessment of liver disease severity based on biochemical and ultrasonographic findings, while transient elastography and baseline liver biopsy were unavailable for some patients. Nevertheless, we can with a reasonable probability exclude cirrhosis in our patients. Last, variability in anti‐copper treatment duration among our patients might have introduced additional bias, and with only three treatment‐naïve patients included, it was not possible to statistically compare treated and untreated groups. At that, baseline brain MRI scans were unavailable for most patients and hence we were unable to report whether any of our patients presented with WM signal changes before treatment initiation; this finding would suggest that demyelination precedes WM volume reduction.

In conclusion, our results suggest that diffuse pontocerebellar and lobar WM atrophy are the earliest brain abnormalities occurring before the onset of clinically overt neurological symptoms in WD encouraging the use of brain MRI for the diagnosis and monitoring in WD patients, regardless of the presence of neuropsychiatric symptoms. Future studies should be conducted in treatment‐naïve patients using prospective designs and incorporating MRI techniques sensitive to myelin integrity, such as diffusion tensor imaging, magnetization transfer imaging, ultra‐short echo‐time imaging, and myelin water imaging.

## AUTHOR CONTRIBUTIONS

Parya Rahimi: study planning, MRI analysis and interpretation, and drafting the manuscript. Stanislav Mareček and Petr Sojka: MRI analysis and results interpretation, and revising the article. Radan Brůha and Tomasz Litwin: study planning, patient recruitment and examination, and revising the article. Monika Dezortová and Milan Hájek: study conduct, MRI acquisition, results interpretation, and revising the article. Marta Skowrońska, Łukasz Smoliński, and Petr Urbánek: study conduct, patient recruitment and examination, and revising the article. Petr Dušek: study planning, design, MRI analysis and interpretation, revising the article, and Guarantor.

## FUNDING INFORMATION

This research was supported by projects Nr. LX22NPO5107 (MEYS)—Financed by European Union—Next Generation EU, and by Ministry of Health of the Czech Republic, DRO—IKEM, IN 00023001, MH CZ‐DRO‐VFN64165, and NV15‐25602A.

## CONFLICT OF INTEREST STATEMENT

Stanislav Mareček received funding from the Czech Ministry of Health, project MH CZ‐DRO‐VFN64165, and the National Institute for Neurological Research, Programme EXCELES, ID Project No. LX22NPO5107 (MEYS): Financed by European Union—Next Generation EU; Monika Dezortová and Milan Hájek received funding from the Czech Ministry of Health, grant No. NV15‐25602A and DRO—IKEM, IN 00023001; Petr Dusek received funding from the Czech Ministry of Health, grant No. NV15‐25602A and project MH CZ‐DRO‐VFN64165, National Institute for Neurological Research, Programme EXCELES, ID Project No. LX22NPO5107 (MEYS): Financed by European Union—Next Generation EU and honoraria for presentations from Medis Pharma, Orphalan Limited, and Alexion. None of the funding bodies had any input into the study design or analysis. Parya Rahimi, Radan Brůha, Petr Sojka, Marta Skowrońska, Łukasz Smoliński, Petr Urbánek, and Tomasz Litwin declare that they have no conflict of interest.

## ETHICS STATEMENT

All procedures followed were in accordance with the ethical standards of the responsible committee on human experimentation (institutional and national) and with the Helsinki Declaration of 1975, as revised in 2000 (5). Informed consent was obtained from all patients for being included in the study. The study was approved by the appropriate Institutional Review Boards, the Bioethical Committee of the Institute of Psychiatry and Neurology, Warsaw, No, 24/2016 and the Ethics Committee of the General University Hospital, Prague, No. 110/14.

## Supporting information


**Table S1.** Comprehensive clinical information of Wilson disease patients.

**Table S2.** Mean regional volumes in Wilson disease and controls separately for both centers.
**Table S3.** Mean regional volumes in Wilson disease and controls processed with the neuroHarmonize tool.
**Table S4.** Mean regional volumes in Wilson disease patients treated by D‐Penicillamine and Zinc processed with the neuroHarmonize tool.
**Table S5.** Voxel‐based morphometry analysis of the white matter comparing Wilson disease patients and controls.

## Data Availability

Data from this will be made available on reasonable request to the corresponding author.
