# Supplementary material for: Brain morphometry in hepatic Wilson disease patients
Source: J Inherit Metab Dis. 2024 Nov 19;48(1):e12814. doi: 10.1002/jimd.12814 (PMC11670153; doi:10.1002/jimd.12814)
Supplement: Supplementary file 1 — Table S1. Comprehensive clinical information of Wilson disease patients. [file JIMD-48-0-s001.docx]

| **Supplementary Table 1:** Comprehensive clinical information of Wilson disease patients | | | | | | | | | | | | | | | |
| --- | --- | --- | --- | --- | --- | --- | --- | --- | --- | --- | --- | --- | --- | --- | --- |
| **Code** | **Sex** | **Age at MRI** | **ATP7B mutation** | **First symptoms/ signs** | **K-F rings** | **Liver disease severity** | **Liver morhology findings** | **Treatment duration** | **Pharmaco-therapy** | **UWDRS part II score** | **UWDRS part III score** | **MRI AT  score** | **MRI CD score** | **Child-Pugh score** | **FIB-4 index** |
| PRG01 | M | 37 | p.[Gln7Aspfs*14];[0] | Increased LFT | 0 | S | histology at baseline: non-specific changes; US: severe steatosis (CAP 314 dB/m), no fibrosis (fibroscan 6 kPa) | 19 | D-PEN 900mg | 0 | 1 | 0 | 0 | 5 | 0,79 |
| PRG02 | F | 19 | p.[His1069Gln];[His1069Gln] | Increased LFT, fatigue | 0 | N | US: normal finding (fibroscan 4.3 kPa) | 3 | D-PEN 900mg | 0 | 0 | 0 | 0 | 5 | 0,38 |
| PRG03 | F | 52 | p.[His1069Gln];[Val1217_Leu1218del] | Fatigue, dyspepsia (family screening) | 0 | S | US: steatosis | 42 | Zinc 150 mg | 0 | 4 | 0 | 1 | 5 | 1,03 |
| PRG04 | M | 29 | p.[Asp1047Valfs*74];[His1069Gln] | Increased LFT | n/a | S | US: steatosis, no fibrosis (fibroscan 6.6 kPa) | 24 | Zinc 200 mg | 0 | 5 | 0 | 2 | 5 | 0,52 |
| PRG05 | M | 25 | p.[His1069Gln];[His1069Gln] | Increased LFT | 0 | SF | US: mild steatosis (CAP 246 dB/m), mild fibrosis (fibroscan 9.9 kPa) | 18 | D-PEN 450mg; Zinc 150 mg | 0 | 2 | 0 | 1 | 5 | 0,48 |
| PRG06 | F | 28 | p.[His1069Gln];[His1069Gln] | Increased LFT, abdominal pain | 0 | SF | histology at baseline: steatofibrosis; US: steatofibrosis | 5 | D-PEN 900mg | 0 | 0 | 0 | 0 | 5 | 0,61 |
| PRG08 | M | 37 | p.[Leu1088*];[?] IVS10+2T-C | Increased LFT | 0 | SF | histology at baseline: steatosis; US: steatosis (CAP 299 dB/m), mild fibrosis (fibroscan 7 kPa) | 21 | D-PEN 1200mg | 1 | 2 | 0 | 0 | 5 | 1,25 |
| PRG09 | F | 51 | p.[His1069Gln];[His1069Gln] | Increased LFT (family screening) | n/a | S | US: hepatomegaly and steatosis | 35 | D-PEN 900mg | 1 | 1 | 0 | 0 | 5 | 2,6 |
| PRG10 | M | 27 | p.[His1069Gln];[Ala1135Glnfs*13] | Increased LFT | 0 | N | histology at baseline: incipient cirrhosis; US: normal findings (CAP 229 dB/m; fibroscan 6.7 kPa) | 17 | D-PEN 900mg | 0 | 2 | 0 | 0 | 5 | 0,43 |
| PRG11 | M | 36 | p.[Trp779*];[Val1262Phe] | Increased LFT, fatigue | 0 | N | US: normal finding | 22 | D-PEN 1200mg | 1 | 2 | 0 | 0 | 5 | 1,18 |
| WAR01 | M | 19 | p.[His1069Gln]:[Asp1267Glu] | Increased LFT | 0 | S | US: steatosis | 15 | D-PEN 750mg | 0 | 0 | 0 | 1 | 5 | 0,25 |
| WAR02 | M | 21 | p.[His1069Gln];[His1069Gln] | Increased LFT (family screening) | 0 | S | US: steatosis | *de novo* | - | 0 | 0 | 0 | 1 | 5 | 0,41 |
| WAR03 | M | 38 | p.[Trp779*];[0] | Increased LFT | 0 | N | US: normal finding | 22 | Zinc 180mg | 0 | 0 | 0 | 2 | 5 | 1,7 |
| WAR07 | M | 25 | p.[His1069Gln];[0] | Increased LFT (family screening) | 0 | N | US: normal finding | *de novo* | - | 0 | 0 | 0 | 0 | 5 | 0,66 |
| WAR11 | M | 29 | p.[His1069Gln];[His1069Gln] | Increased LFT | 1 | N | US: normal finding (fibroscan 4.5 kPa) | 11 | D-PEN1000mg | 0 | 0 | 0 | 1 | 5 | 0,53 |
| WAR16 | F | 19 | p.[His1069Gln];[Gln355*] | Increased LFT | 0 | S | US: steatosis | 14 | Zinc 180mg | 0 | 0 | 0 | 1 | 5 | 0,38 |
| WAR17 | M | 28 | p.[His1069Gln];[His1069Gln] | Increased LFT, abdominal pain (family screening) | 0 | S | US: hepatomegaly | 25 | Zinc 180mg | 0 | 0 | 0 | 1 | 5 | 0,35 |
| WAR18 | F | 26 | p.[His1069Gln];[His1069Gln] | Increased LFT (family screening) | 0 | N | US: normal finding | 21 | Zinc 180mg | 0 | 0 | 0 | 1 | 5 | 0,42 |
| WAR20 | F | 23 | p.[His1069Gln];[His1069Gln] | Increased LFT, hemolytic anemia | 1 | S | US: increased liver echogenity (steatosis) | 0,5 | D-PEN 1000mg | 0 | 0 | 0 | 2 | 5 | 0,81 |
| WAR22 | M | 32 | p.[His1069Gln];[His1069Gln] | Increased LFT | 0 | S | US: hepatomegaly and steatosis | 2 | Zinc 180mg | 0 | 0 | 0 | 1 | 5 | 0,76 |
| WAR23 | F | 39 | p.[His1069Gln];[0] | Increased LFT | 0 | S | US: steatosis | 19 | Zinc 180mg | 0 | 0 | 0 | 1 | 5 | 0,58 |
| WAR25 | M | 25 | p.[His1069Gln];[Arg969Gln] | Increased LFT (family screening) | 0 | N | US: normal finding | 1 | Zinc 180mg | 0 | 0 | 0 | 0 | 5 | 0,48 |
| WAR26 | M | 26 | p.[His1069Gln];[His1069Gln] | Increased LFT | 0 | SF | US: hepatomegaly, steatosis, severe fibrosis (fibroscan 14.7 kPa) | 3 | Zinc 180mg | 0 | 0 | 0 | 1 | 5 | 0,55 |
| WAR27 | F | 22 | p.[His1069Gln];[His1069Gln] | Increased LFT, abdominal pain, ammenorhhea | 0 | S | US: hepatomegaly, steatosis, no fibrosis (fibroscan 6.8 kPa) | *de novo* | - | 0 | 0 | 0 | 0 | 5 | 0,42 |
| WAR28 | F | 21 | p.[His1069Gln];[0] | Increased LFT, fatigue | 0 | S | US: hepatomegaly, increased liver echogenity (steatosis) | 2 | Zinc 180mg | 0 | 1 | 0 | 2 | 5 | 0,58 |
| WAR30 | F | 25 | p.[His1069Gln];[0] | Increased LFT, abdominal pain | 0 | S | US: hepatomegaly, steatosis, no fibrosis (fibroscan 5.2 kPa) | 4 | D-PEN 1000mg | 0 | 0 | 0 | 0 | 5 | 0,45 |
| M, male; F, female; LFT, liver function tests; n/a, not available; S, steatosis; F, fibrosis; N, normal liver morphology; US, ultrasound; CAP, Controlled Attenuation Parameter; UWDRS, Unified Wilson Disease Rating Scale; MRI AT, magnetic resonance imaging acute toxicity; MRI CD, magnetic resonance imaging chronic damage; *de novo*, newly diagnosed treatment-naive patient | | | | | | | | | | | | | | | |
