# Supplementary material for: Brain morphometry in hepatic Wilson disease patients
Source: J Inherit Metab Dis. 2024 Nov 19;48(1):e12814. doi: 10.1002/jimd.12814 (PMC11670153; doi:10.1002/jimd.12814)
Supplement: Supplementary file 2 — Table S2. Mean regional volumes in Wilson disease and controls separately for both centers. Table S3. Mean regional volumes in Wilson disease and controls processed with the neuroHarmonize tool. Table S4. Mean regional volumes in Wilson disease patients treated by D‐Penicillamine and Zinc processed with the neuroHarmonize tool. Table S5. Voxel‐based morphometry analysis of the white matter comparing Wilson disease patients and controls. [file JIMD-48-0-s002.docx]

| **Supplementary Table 2:** Mean regional volumes in Wilson disease and controls separately for both centers | | | | |
| --- | --- | --- | --- | --- |
|  | Prague | | Warsaw | |
|  | WD (n=10) | controls (n=16) | WD (n=16) | controls (n=12) |
| age | 34.0 ± 10.9 | 31.4 ± 8.5 | 26.1 ± 6.0 | 34.3 ± 10.0 |
| BPF (%) | 0.80 ± 0.03 | 0.81 ± 0.02 | **0.81 ± 0.04** | **0.84 ± 0.04** |
| Nucleus Accumbens | 1.0 ± 0.1 | 1.0 ± 0.2 | 1.1 ± 0.2 | 1.1 ± 0.1 |
| Amygdala | 3.4 ± 0.3 | 3.5 ± 0.3 | 3.2 ± 0.4 | 3.3 ± 0.4 |
| Caudate | 6.6 ± 0.7 | 7.3 ± 0.8 | 7.7 ± 1.0 | 7.4 ± 0.9 |
| Cerebellar Cortex | 109.0 ± 8.0 | 112.5 ± 6.8 | 115.8 ± 11.1 | 117.9 ± 9.0 |
| Cerebellar White Matter | 26.2 ± 4.3 | 29.2 ± 3.3 | **26.9 ± 3.3** | **28.8 ± 2.8** |
| Hippocampus | 8.8 ± 0.8 | 8.5 ± 0.5 | 8.5 ± 0.6 | 8.1 ± 0.5 |
| Midbrain | 5.9 ± 0.4 | 6.1 ± 0.4 | **6.3 ± 0.3** | **6.5 ± 0.4** |
| Globus Pallidus | 3.8 ± 0.5 | 4.0 ± 0.2 | **3.9 ± 0.4** | **4.2 ± 0.4** |
| Pons | **13.0 ± 1.7** | **14.6 ± 1.5** | **14.3 ± 1.3** | **15.6 ± 1.4** |
| Putamen | 9.8 ± 1.0 | 9.7 ± 0.8 | 10.3 ± 1.3 | 10.1 ± 1.0 |
| Superior Cerebellar Peduncle | 0.2 ± 0.1 | 0.3 ± 0.0 | 0.3 ± 0.0 | 0.3 ± 0.0 |
| Thalamus | 14.9 ± 1.3 | 15.5 ± 0.9 | **15.1 ± 1.1** | **15.4 ± 1.2** |
| Mean values ± standard deviations are reported; except of BPF all values are shown as TIV-corrected volumes in [cm^3^]; significant differences between Wilson disease patients and controls adjusted for age and sex at each center are marked with **BOLD** font. | | | | |

| **Supplementary Table 3:**  Mean regional volumes in Wilson disease and controls processed with the neuroHarmonize tool | | | |
| --- | --- | --- | --- |
|  | WD | Controls | p-value |
| Nucleus Accumbens | 1.1 ± 0.2 | 1.0 ± 0.2 | 0.74 |
| Amygdala | 3.3 ± 0.4 | 3.4 ± 0.3 | 0.27 |
| Caudate | 7.2 ± 1.0 | 7.4 ± 0.8 | 0.33 |
| Cerebellar Cortex | 112.9 ± 9.7 | 115.1 ± 7.6 | 0.10 |
| Cerebellar White Matter | 26.8 ± 3.6 | 29.0 ± 3.0 | **0.02** |
| Hippocampus | 8.6 ± 0.7 | 8.3 ± 0.5 | 0.07 |
| Midbrain | 6.1 ± 0.3 | 6.3 ± 0.4 | **0.05** |
| Globus Pallidus | 3.9 ± 0.4 | 4.1 ± 0.3 | 0.06 |
| Pons | 13.7 ± 1.4 | 15.1 ± 1.4 | **<0.001** |
| Putamen | 10.1 ± 1.1 | 9.9 ± 0.9 | 0.67 |
| Superior Cerebellar Peduncle | 0.2 ± 0.0 | 0.3 ± 0.0 | 0.06 |
| Thalamus | 15.0 ± 1.1 | 15.4 ± 1.0 | **0.05** |
| Mean values ± standard deviations are reported; values are shown as volumes harmonized using the neuroHarmonize tool in [cm^3^]; significant differences between Wilson disease patients and controls adjusted for age and sex are marked with **BOLD** font. | | | |

| **Supplementary Table 4:**  Mean regional volumes in Wilson disease patients treated by D-Penicillamine and Zinc processed with the neuroHarmonize tool | | | |
| --- | --- | --- | --- |
|  | D-PEN (n=11) | Zinc (n=11) | p-value |
| Age (years) | 30.1 ± 9.5 | 30.4 ± 9.5 | 0.95 |
| Sex Females/Males, n | 5/6 | 5/6 | 0.99 |
| Center PRG/WAR, n | 7/4 | 2/9 | 0.03 |
| BPF (%) | 0.80 ± 0.03 | 0.82 ± 0.03 | 0.18 |
| Nucleus Accumbens | 1.0 ± 0.1 | 1.1 ± 0.2 | 0.13 |
| Amygdala | 3.3 ± 0.3 | 3.3 ± 0.4 | 0.83 |
| Caudate | 6.9 ± 0.7 | 7.4 ± 1.1 | 0.17 |
| Cerebellar Cortex | 112.2 ± 8.6 | 110.0 ± 8.8 | 0.53 |
| Cerebellar White Matter | 26.3 ± 3.7 | 27.1 ± 3.4 | 0.61 |
| Hippocampus | 8.6 ± 0.7 | 8.7 ± 0.7 | 0.52 |
| Midbrain | 6.0 ± 0.3 | 6.1 ± 0.3 | 0.41 |
| Globus Pallidus | 3.8 ± 0.4 | 4.0 ± 0.4 | 0.43 |
| Pons | 13.5 ± 1.4 | 13.9 ± 1.5 | 0.45 |
| Putamen | 9.8 ± 1.0 | 10.2 ± 1.1 | 0.40 |
| Superior Cerebellar Peduncle | 0.2 ± 0.1 | 0.2 ± 0.0 | 0.86 |
| Thalamus | 14.9 ± 1.2 | 15.0 ± 1.2 | 0.87 |
| Mean values ± standard deviations are reported; values are shown as volumes harmonized using the neuroHarmonize tool in [cm^3^]; p-values are adjusted for age and sex. | | | |

| **Supplementary Table 5:** Voxel-based morphometry analysis of the white matter comparing Wilson disease patients and controls | | | |
| --- | --- | --- | --- |
| T-value | Volume [mm^3^] | XYZ coordinates [mm] | Structures |
| 5.2 | 31905 | -33×22×34 | Cortico Spinal Left; Corpus Callosum Left; Cingulum Left; Internal Capsule Left; Arcuate Posterior Segment Left; Arcuate Anterior Segment Left; Long Segment Left; Inferior Occipito-Frontal Fasciculus Left; Cortico-Ponto-Cerebellum Left |
| 4.9 | 5877 | 32×-16×-6 | Optic Radiations Right; Inferior Occipito-Frontal Fasciculus Right; Inferior Longitudinal Fasciculus Right; Fornix Right; Cingulum Right; Corpus Callosum Right; Anterior Commissure Right |
| 4.8 | 33261 | 35×31×30 | Cortico Spinal Right; Corpus Callosum Right; Cingulum Right; Arcuate Anterior Segment Right; Long Segment Right; Internal Capsule Right; Cortico-Ponto-Cerebellum Right; Arcuate Posterior Segment Right |
| 4.5 | 3873 | 29×-61×15 | Corpus Callosum Right; Inferior Longitudinal Fasciculus Right; Inferior Occipito-Frontal Fasciculus Right; Internal Capsule Right; Arcuate Posterior Segment Right; Optic Radiations Right |
| 4.4 | 1812 | -34×-17×-7 | Inferior Occipito-Frontal Fasciculus Left; Fornix Left; Inferior Longitudinal Fasciculus Left; Optic Radiations Left; Corpus Callosum Left; Anterior Commissure Left |
| 4.4 | 402 | -37×-17×-20 | Inferior Longitudinal Fasciculus Left |
| 4.2 | 27087 | 9×-61×-46 | Internal Capsule Right; Cortico-Ponto-Cerebellum Left; Cortico-Spinal Left; Internal Capsule Left; Cortico-Spinal Right; Cortico-Ponto-Cerebellum Right; Inferior Cerebellar Pedunculus Left; Superior Cerebelar Pedunculus Left |
| 3.3 | 1832 | -28×-79×18 | Corpus Callosum Left; Inferior Longitudinal Fasciculus Left; Optic Radiations Left; Internal Capsule Left |
| 3.3 | 430 | 23×-54×1 | Inferior Occipito-Frontal Fasciculus Right |
| 3.2 | 143 | -51×2×16 | Long Segment Left; Arcuate Anterior Segment Left |
| 3.1 | 39 | -10×31×38 | Corpus Callosum Left |
| 3.1 | 188 | 49×-31×-16 | Inferior Longitudinal Fasciculus Right |
| 3.1 | 587 | 14×-74×-37 | Superior Cerebelar Pedunculus Right; Cortico-Ponto-Cerebellum Left |
| 3.0 | 62 | -17×-54×-26 | Cortico-Ponto-Cerebellum Right |
| 2.8 | 82 | -15×0×50 | Cortico Spinal Left; Corpus Callosum Left; Internal Capsule Left; Cingulum Left |
| Significant clusters (thresholded at P_FWE_ < 0.05) for the contrast controls > WD are shown;  T-values and MNI coordinates at peak levels as well as involved anatomical structures are listed | | | |
